# Policy transmission of “Healthy China 2030” on community health curriculum reform in higher vocational medical education

**DOI:** 10.3389/fpubh.2026.1772966

**Published:** 2026-03-10

**Authors:** Jun Zhang, Jiao-Yang Nie

**Affiliations:** School of Public Health and Health Management, Henan Medical College, Zhengzhou, China

**Keywords:** community health education, curriculum reform, health policy transmission, health workforce development, policy implementation

## Abstract

This study examines how China’s “Healthy China 2030” policy influences community health curriculum reform in higher vocational medical education through systemic policy transmission mechanisms across governance levels. Employing a qualitative document analysis design with cross-sectional data collection guided by Walt and Gilson’s policy triangle model, this study analyzed 13 national, provincial, and institutional policy documents produced between 2016 and 2025 to map policy development from strategic planning to operational deployment. Based on descriptive document analysis, the findings suggest that the observed policy transmission pattern involves three main mechanisms of content adaptation, language simplification, and contextual integration, facilitating the translation of national health goals into institutional implementation while maintaining long-term strategic consistency. The research identifies an adaptive pattern of transmission beyond sequential assumption of implementation, and a consistent linguistic evolution from strategic abstract direction through to operational activity in the structures of governance. Institutional response reporting indicates varying implementation strategies by organizational context and capacity, where institutions interpret policy objectives into varying mechanisms such as curriculum alignment, faculty development, and building of community partnerships. Preliminary analysis of follow-up reporting indicates institutional development in aligning education practice with policy objectives, although measurement of overall effectiveness is constrained by heterogeneous assessment practice. The analysis provides empirical support for the observation that policy implementation appears associated with active content adjustment rather than stereotypical compliance, suggesting that policymakers should consider flexible models of implementation that accommodate institutional diversity while maintaining strategic integration. The findings offer preliminary insights into adaptive implementation processes in health education policy transmission as identified through document-based policy analysis.

## 1 Introduction

Global health systems face mounting pressure to prepare health professionals capable of addressing emerging healthcare challenges, prompting widespread recognition that traditional medical curricula require fundamental reform (1). These developments have generated debate around restructuring medical education curriculum toward greater responsiveness to population health priorities and healthcare system requirements.

China’s domestic reform effort in healthcare itself is a classic example of these worldwide efforts at change with its own sweeping “Healthy China 2030” plan for an all-encompassing health system for more than 1.4 billion people (2). China’s medical education system has faced significant challenges, particularly as the COVID-19 pandemic exposed gaps between education performance and public health preparedness (3). This environment has encouraged policy-oriented change toward establishment of a health workforce, that is, developing health care capacity (4). Building the community health workforce is one of the key health system strengthening elements, and this calls for training institutions to reform curricula in line with shifting professional competency needs (5). Tertiary vocational medical education is increasingly taking part in the training of front-line health professionals who deliver core health care in a range of community settings (6, 7).

Contemporary literature illustrates significant knowledge gaps in our understanding of how health policy influences medical education reform at an institutional level. The majority of research conducted so far has either described policy content or attempted to measure educational outcomes without adequately addressing the processes through which policies are translated into curriculum change (8, 9). Traditional approaches to medical education evaluation do not tend to capture dynamic interaction between policy implementation and institutional response (10, 11). The absence of general frameworks for analyzing policy-driven education reform impedes the development of evidence-based prescriptions for consolidating health workforce development programs (12). In particular, few studies have traced the linguistic and structural evolution of policy texts as they traverse governance hierarchies from national formulation through provincial interpretation to institutional implementation.

This study addresses these knowledge gaps by examining how “Healthy China 2030” policy influences community health curriculum change in higher vocational medical colleges based on systematic in-depth document analysis. The paper employs a qualitative document analysis approach guided by thematic content analysis for studying policy transmission mechanisms and examining implementation patterns across different institutional contexts. Through systematic review of national, provincial, and institutional policy documents, this study contributes to the global evidence on how national health policy may shape medical education reform and health workforce preparedness to address contemporary public health needs. Specifically, this study aims to trace policy content evolution across three governance levels, identify transmission mechanisms facilitating policy adaptation, and document institutional implementation responses and preliminary outcomes.

## 2 Theoretical framework

### 2.1 Walt and Gilson’s policy triangle framework

Walt and Gilson’s policy triangle analysis model has been among the most popular analytical models for health policy processes conceptualization since its evolution in the 1990s (13). The analytical framework addresses the traditional shortcomings of health policy studies, which have long focused nearly entirely on policy content with no consideration of how context, process, and actors significantly shape policy outcomes (9). The model envisages health policy analysis in terms of four interrelated dimensions that interact with one another to shape policy formulation and implementation. Content includes the precise policy goals, strategies, rules, and guidelines that determine what the policy attempts to accomplish. Context implies the general systemic factors comprising political, economic, social, and cultural conditions that form the environment in which policies are created and implemented. Process examines how policies are initiated, developed, negotiated, communicated, and evaluated throughout their lifecycle. Actors refer to the influential individuals, groups, organizations, and institutions that participate in or influence the policy process at various levels.

Its longevity is attested to by its common usage within both policy and geographic contexts (14). Widespread application of the policy triangle across a range of health issues, from infectious disease control to health system reforms, has been reported in systematic reviews in recent times, most notably of all in low- and middle-income countries where multifaceted policy problems demand sophisticated analytic approaches (15). But later scholars noted the necessity of methodological refinements to make the framework analytically more accurate and practically applicable. Later developments have centered on conceptual clarification of the sides of the triangle, offering researchers more specific prescriptions for undertaking detailed policy analyses (13). The model’s power is in the realization that policy making is an intrinsically interactive process in which all four dimensions interact with each other dynamically, and not as independent variables. It is this synergistic integration that makes the model extremely relevant to the evaluation of multifaceted policy interventions like health workforce development programs, where various stakeholders, varied contexts, and complicated implementation procedures converge to produce educational reform results.

While the policy triangle encompasses four dimensions, this study focuses primarily on content and process dimensions given the document-based methodology. Context analysis requires examination of broader political-economic conditions that extend beyond documentary evidence, and comprehensive actor analysis necessitates interview or survey methods to capture stakeholder perspectives and power dynamics. These limitations are acknowledged, and the analytical focus on content transmission and process mechanisms represents a deliberate methodological choice aligned with available data sources.

### 2.2 Policy transmission theory

Policy transmission theory provides universal insights into the transmission of policies from formulation to implementation at organizational levels and institutional environments. The conventional policy analysis recognizes that policy idea to practice is a complex process of interpretation, adaptation, and translation as policies move through different stakeholders and different levels of institutional capacities (16). This theoretical framework challenges the assumption of linear policy implementation, with a view to the fact that policy is always likely to be modified at every organizational level through which it must be taken. Transmission is most complicated in health systems where policies are required to pass through national, regional, and local government levels and get accustomed to varying professional cultures and institutional agendas (17). Present policy transmission theories emphasize the dynamic nature of the process by which policies are not transmitted in wholes but are re-made by interactional processes between policy intentions and contexts of implementation.

Multi-level systems of governance have increasingly been used in outlining the manner in which policies function at more than one level of power and influence (18). Such systems recognize that leadership in contemporary health policy involves complicated systems of actors functioning at more than a one-level view, from national policy makers to frontline practitioners, each with their own interpretation and boundary of the policy implementation process. Institutional adaptation is the final process of significance in understanding how institutions reassess their structures, processes, and practices in relation to policy requirements without compromising their constituent roles and identities (16). Scholarship describes how successful transmission of policy at times depends on the ability of institutions to interpret policy requirements specifically in their particular contexts rather than adhering to prescribed steps. Such adaptive capacity is most urgently needed within tertiary learning settings where universities must navigate pedagogic autonomy, policy adherence, and limited resources (19). Such transmission mechanisms explain why policies with almost similar content have highly divergent impacts in disparate institutional settings, and provide valuable lessons for policy design and implementation strategies to be optimized.

## 3 Methods

### 3.1 Study design

**Figure 1:**
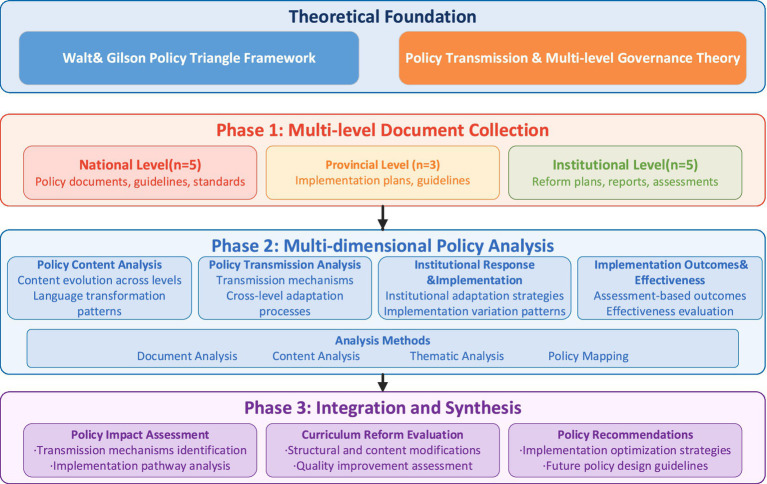
Research design framework.

This study employs a qualitative cross-sectional document analysis design to examine the relationship between “Healthy China 2030” policy and community health curriculum reform in higher vocational medical education institutions. As illustrated in Figure 1, the research follows a sequential three-stage analytical framework: (1) multi-level document collection across national, provincial, and institutional governance levels, (2) multi-dimensional policy analysis encompassing content evolution, transmission mechanisms, institutional responses, and implementation outcomes, and (3) integration and synthesis of findings into policy transmission patterns and reform evaluation. Building on established qualitative policy analysis approaches (20), this research combines systematic document retrieval with multi-dimensional policy analysis to capture policy transmission processes as documented across governance levels. This design recognizes that comprehensive policy analysis requires integrated examination of content evolution, transmission processes, institutional responses, and implementation documentation as constituent parts of a coherent analytical framework.

The research design integrates Walt and Gilson’s policy triangle framework with policy transmission theory to structure analysis across four complementary dimensions: policy content evolution, transmission mechanisms, institutional response patterns, and implementation documentation. These four analytical dimensions operationalize primarily the content and process components of Walt and Gilson’s model, consistent with the analytical rationale discussed in Section 2.1. This multi-dimensional approach, consistent with contemporary qualitative policy research methodologies (21), enables systematic examination of how national policy directions are reflected in institutional educational practices as documented across governance levels. Importantly, this study employs exclusively qualitative analytical methods; no statistical analyses were conducted, as the study’s analytical objectives and the interpretive nature of policy document analysis are best addressed through qualitative approaches.

As a cross-sectional document analysis, this study enables identification of patterns and associations between policy formulation and institutional implementation but does not permit causal inference. The observed alignment between national policy objectives and institutional curriculum changes may reflect independent institutional initiatives responding to similar environmental pressures, coincidental timing, or policy influence operating through the documented transmission channels. Establishing causal relationships would require longitudinal designs with baseline measurements or comparative analysis with control institutions not exposed to policy interventions. Accordingly, this study focuses on documenting observable patterns of policy content evolution and institutional response rather than attributing causation, providing a descriptive foundation upon which future explanatory research may build.

### 3.2 Data collection

Documents were collected across three governance levels through criterion-based purposive selection, targeting official policy texts directly relevant to community health curriculum reform within China’s health education system. As summarized in Table 1, 13 policy documents spanning 2016–2025 were selected to trace how “Healthy China 2030” policy objectives are reflected in provincial and institutional educational practices. National-level documents (n = 5) establish the policy foundation, including the “Healthy China 2030” Planning Outline and Health Commission guidelines on medical education standards and community health service development plans. Provincial-level documents (n = 3) capture regional adaptation of national directives, while institutional-level documents (n = 5) provide evidence of ground-level curriculum reform responses across different document types, including reform plans, implementation reports, and assessment documents.

**Table 1:** Policy document collection framework.

Governance level	Document type	Number (n)	Time period	Key content focus	Data collection criteria
National	“Healthy China 2030” Strategic Blueprint	1	2016–2030	National health strategy, workforce development goals	Official policy document with curriculum implications
National	Health commission guidelines	2	2018–2024	Medical education standards, community health service development	Direct relevance to community health education
National	Education curriculum standards	1	2019–2025	Vocational education reform, curriculum requirements	Specific mention of health workforce training
National	Vocational reform policies	1	2020–2024	Higher vocational education transformation	Community health program specifications
Provincial	Implementation plans	1	2018–2023	Regional policy adaptation, local contextualization	Provincial-level curriculum reform initiatives
Provincial	Educational guidelines	1	2019–2024	Institutional compliance requirements, quality standards	Direct implementation guidance for institutions
Provincial	Workforce development strategies	1	2020–2025	Regional health workforce planning, training priorities	Community health workforce focus
Institutional	Reform plans	2	2019–2024	Curriculum restructuring, program modifications	Evidence of policy-driven curriculum changes
Institutional	Implementation reports	2	2020–2024	Reform progress, compliance measures, outcomes	Documented institutional responses to policy
Institutional	Assessment documents	1	2021–2024	Quality evaluation, student competency assessment	Measurable curriculum reform impacts

This framework demonstrates the systematic collection of policy documents across three governance levels, encompassing 13 documents that trace policy transmission from national strategic formulation through provincial adaptation to institutional implementation. Health Commission Guidelines include both medical education standards and community health service development plans, reflecting the integrated approach to health workforce development within the “Healthy China 2030” framework. Document selection followed criterion-based purposive selection procedures, prioritizing official policy texts with direct relevance to community health curriculum reform, while document availability across governance levels determined the final scope of the analytical sample.

The selection process was guided by the following inclusion criteria: selected texts were required to (1) be officially issued by government agencies or accredited educational institutions between 2016 and 2025; (2) contain explicit reference to community health curriculum reform, health workforce development, or professional competency standards; and (3) be issued within the policy context of China’s health system reform initiated by the “Healthy China 2030” framework or its derivative directives. Documents without direct relevance to community health education were excluded. The timeframe covers the policy implementation period from the release of the “Healthy China 2030” Planning Outline (2016) through subsequent reform phases, enabling examination of policy documents produced at different stages of the reform process. Institutional-level document sources were particularly valuable in documenting how national policy objectives are reflected in curriculum reforms at ground-level implementation, with reform proposals, implementation reports, and evaluation reports offering evidence of institutional transformation processes, consistent with recommendations for systematic data source identification in health professions education research (22).

National-level documents included the “Healthy China 2030” Planning Outline (2016), guidelines on medical education reform from the State Council (2017), primary healthcare capacity building directives from the National Health Commission (2018), vocational education quality improvement plans (2020), and professional teaching standards for higher vocational medical education (2019). Provincial-level documents were collected from one eastern coastal province, including the provincial implementation plan for “Healthy China 2030,” health workforce development planning, and guidelines for medical and health program development in higher vocational education. Institutional documents were obtained from three higher vocational medical colleges within the same province, encompassing curriculum reform plans, implementation progress reports, and quality assessment reports. The province was selected based on accessibility of complete provincial and institutional policy documentation and the presence of documented health education reform initiatives under the “Healthy China 2030” framework. Document selection was constrained by the availability of official policy texts meeting the inclusion criteria; however, the 13 selected documents represent a purposive analytical sample rather than an exhaustive collection of all relevant policy texts. The selection prioritized documents that collectively trace the complete policy chain from national strategic formulation through provincial adaptation to institutional implementation, providing sufficient analytical coverage across the four dimensions outlined in Section 3.1. The selection of institutions from one eastern coastal province may introduce geographic bias, as implementation patterns in less-developed regions may differ; this limitation is addressed in Section 5. Provincial and institutional sources are anonymized to protect confidentiality; full document details are available from the corresponding author upon reasonable request.

### 3.3 Analysis framework

This study employs thematic content analysis within the document analysis framework established in Section 3.1, as policy transmission manifests through linguistic and substantive changes in official texts that can be systematically identified across organizational levels. This approach allows for the identification of policy language evolution patterns that would otherwise be elusive using other approaches, while thematic analysis of institutional documents identifies repeated patterns of institutional response and implementation strategies. Within this analytical framework, content tracking follows selected policy elements across national, provincial, and institutional documents, while cross-level comparison charts how policy messages are adapted as they pass through transmission stages. The analysis is structured by Walt and Gilson’s policy triangle framework, with the content and process dimensions providing the primary analytical lens as outlined in Section 2.1.

The analysis operationalizes two of Walt and Gilson’s four dimensions: content (tracking policy language evolution across governance levels) and process (examining transmission mechanisms and institutional responses). The context dimension is addressed descriptively through acknowledgment of China’s health system reform background, while actor analysis falls outside the scope of document-based methodology and represents an area for future research involving stakeholder interviews.

The analytical process involved three stages: (1) initial reading and familiarization with all 13 documents to develop preliminary understanding of policy content and transmission patterns; (2) systematic coding of policy language, implementation strategies, and outcome indicators using a deductive coding framework structured around the four analytical dimensions identified in Section 3.1: policy content evolution, transmission mechanisms, institutional response patterns, and implementation documentation; and (3) cross-level comparison to identify transmission patterns and adaptation mechanisms across governance levels. To ensure analytical consistency, both researchers independently coded one document from each governance level (3 of 13 documents) using the deductive coding framework. Coding discrepancies were discussed and resolved through consensus, and the coding framework was refined accordingly. The primary researcher then applied the finalized framework to all 13 documents, with the second researcher reviewing and verifying the complete coding results.

The analytical approach incorporates triangulation across multiple document sources and governance levels to strengthen analytical consistency, consistent with established standards of program evaluation in health professions settings (23). This approach does not capture stakeholder perceptions or direct experience of implementation, which represents a priority area for future mixed-methods research.

## 4 Results

### 4.1 Policy content analysis

Policy content analysis reveals patterns of content evolution across governance levels, documenting how abstract national health objectives are expressed differently as policy documents move from strategic planning to institutional practice. As Table 2 demonstrates, national-level strategic formulations such as “comprehensive community health service capacity building” are progressively specified through provincial-level adaptation and appear in institutional documents as concrete directives for curriculum integration and competency-based evaluation approaches. This pattern of progressive specification is consistent with the policy transmission framework outlined in Section 2, where policy intentions are maintained but adapted to fit organizational contexts and operational conditions. The analysis suggests that the observed policy transmission pattern involves systematic content modification rather than hierarchical replication, with each governance level contributing contextually relevant specification to the policy content. Provincial documents serve as intermediary translations, interpreting broad national agendas into regionally relevant guidance applicable within existing education systems. The documented content evolution indicates a pattern of language development from strategic abstractions to operational guidance, though whether this pattern reflects intentional policy design or emergent institutional adaptation warrants further investigation beyond documentary evidence.

**Table 2:** Policy content evolution across governance levels.

Policy element	National level expression	Provincial level adaptation	Institutional level implementation
Community health workforce development	“Strengthen primary healthcare through comprehensive community health service capacity building”	“Develop community-oriented health professional training programs addressing regional population health needs”	“Integrate community health practice components into clinical education through local healthcare partnerships”
Curriculum reform objectives	“Transform health professional education to support Healthy China 2030 strategic goals”	“Align medical education curricula with provincial health system requirements and workforce development priorities”	Specific curriculum reform objectives not detailed in available institutional documents
Professional competency standards	“Cultivate health professionals with comprehensive primary healthcare capabilities and community service orientation”	Competency framework specifications not provided in provincial documentation	“Implement competency-based assessment methods including practical skills evaluation and community health project requirements”
Educational quality assurance	“Establish national evaluation frameworks ensuring educational quality and professional competency development”	“Create provincial monitoring mechanisms with standardized assessment criteria and institutional compliance requirements”	“Develop internal quality management systems including curriculum evaluation protocols and student outcome tracking”
Implementation guidance	“Achieve comprehensive health education reform supporting national health development objectives”	“Complete curriculum alignment and institutional capacity building within designated reform implementation period”	Implementation timeline details vary across institutional documents with limited specification
Resource development	“Enhance investment in health education infrastructure and professional workforce development capacity”	Resource allocation strategies not systematically documented in available provincial materials	“Prioritize resource allocation for curriculum materials development, faculty skill enhancement, and clinical practice site establishment”

Analysis based on available policy documents across three governance levels. Missing information reflects varying document focus and availability across policy domains and governance levels. Findings represent documented evidence rather than comprehensive policy coverage, acknowledging limitations inherent in document-based analysis.

### 4.2 Policy transmission analysis

**Figure 2:**
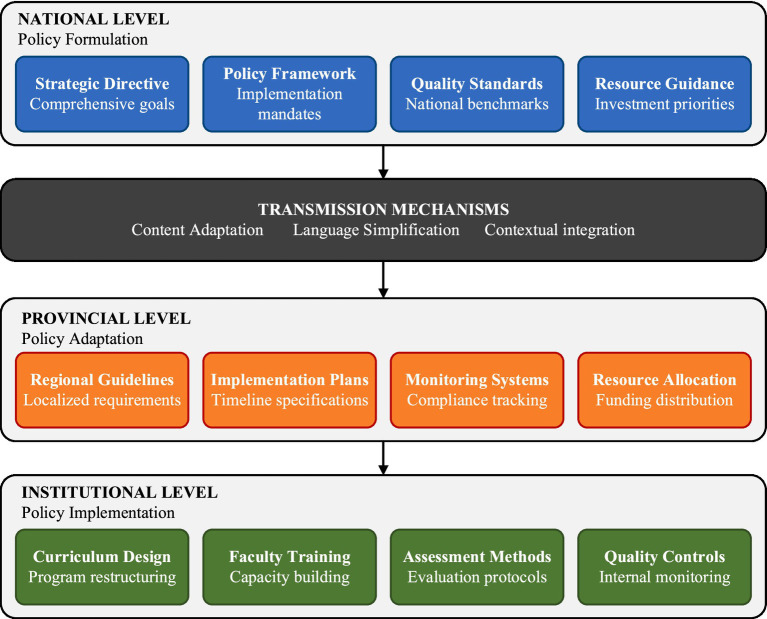
Policy transmission mechanisms across governance levels. This Figure illustrates the three identified transmission patterns (content adaptation, language simplification, and contextual integration) operating across the three governance levels. Each governance level shows four documented functional areas (e.g., at the national level: strategic directives, policy frameworks, quality standards, and resource guidance), with downward arrows indicating the documented direction of policy content specification from national formulation (top) through provincial adaptation (middle) to institutional implementation (bottom).

Policy transmission analysis identifies sophisticated patterns of adaptation across governance levels through which national strategic guidelines are progressively specified toward operational institutional practice, as illustrated in Figure 2. Three transmission patterns, characterized as content adaptation, language simplification, and contextual integration, are identified in the documentary evidence, appearing to facilitate policy specification across governance hierarchies while maintaining strategic coherence. National-level policy frameworks are progressively elaborated across provincial and institutional levels, with each level contributing context-specific detail to the policy content. Provincial-level documents indicate not only content adaptation but also the development of monitoring systems connecting strategic objectives with operational requirements. The observed transmission process suggests that policy specification across governance levels involves systematic content adaptation that maintains fundamental objectives while accommodating contextual constraints, rather than mechanistic replication of policy language.

At the institutional level, policy content appears in its most operationally specific form, with national standards expressed through curriculum design specifications, faculty development requirements, and quality assurance procedures. This progression from national formulation to institutional implementation is illustrated in Figure 2, highlighting both consistency in core policy messaging and context-specific adaptation at each level. The documentary evidence suggests that institutional implementation patterns are associated with provincial-level planning and resource allocation, though the nature and direction of this relationship cannot be established through document analysis alone.

### 4.3 Institutional response and implementation

Documentation of institutional response reveals both common patterns and notable variations in implementation approaches across the three institutions, with differences potentially reflecting distinct organizational contexts and documentation practices. Table 3 presents how different document types provide complementary perspectives on institutional adaptation processes: reform plans focus on structural planning, implementation reports document operational progress where recorded, and assessment documents emphasize outcome measurement with limited attention to process description. Available evidence indicates that curriculum integration approaches range from modular additions to broader program restructuring, though the scope of these efforts varies across institutional documents. Community partnership development represents the most inconsistently documented area, with significant gaps suggesting either differing implementation approaches or differential reporting practices across institutions.

**Table 3:** Institutional implementation strategies based on document analysis.

Implementation focus	Reform plan, institution A (2021)	Implementation report, institution B (2022)	Assessment document, institution C (2023)
Curriculum integration approach	“Establish community health practice modules as separate course components within existing clinical training framework”	“Integration of community health content across core courses shows completion in selected programs”	Assessment focuses on learning outcomes rather than curriculum structure
Faculty development strategy	“Organize training workshops for faculty on community health teaching methods”	Faculty development initiatives mentioned but details not specified in implementation reporting	“Faculty competency in community health education assessed through peer evaluation and student feedback”
Community partnership development	Partnership development not detailed in reform planning documents	“Partnerships established with community organizations and primary care providers during implementation phase”	“Community partnership effectiveness measured through student placement feedback”
Quality monitoring mechanisms	“Implement regular reporting systems to provincial authorities with compliance documentation and progress tracking”	“Internal evaluation protocols developed combining external requirements with institutional improvement initiatives”	“Assessment framework demonstrates compliance with provincial quality indicators”
Implementation timeline	“Execute implementation in two-year phases: preparatory curriculum development, pilot program launch”	Timeline adherence reported but specific phases not detailed in available documentation	Assessment conducted during post-implementation phase but timeline not specified

This table presents implementation strategies documented across three institutions, with each column representing a different document type from a different institution, reflecting the varying documentation available from each source. Missing information reflects document-specific focus areas and availability constraints inherent in institutional documentation practices.

More broadly, document analysis suggests that institutional implementation involves selective emphasis on different strategic priorities, though the evidence base remains fragmented across document types and institutional contexts. Faculty development receives inconsistent documentation attention, with some institutions providing detailed mentorship program descriptions while others offer limited evidence of systematic capacity building initiatives. This pattern indicates that institutions prioritize different implementation dimensions based on their documented capabilities, though available documentation does not permit assessment of how these varying priorities relate to implementation outcomes.

### 4.4 Implementation outcomes and effectiveness

Implementation outcome analysis indicates that assessment documentation practices vary substantially across institutions, creating challenges for comprehensive effectiveness evaluation. Table 4 illustrates that available assessment documents provide fragmentary rather than systematic evidence of implementation outcomes, with institutions selectively documenting different effectiveness dimensions based on their evaluation priorities and documentation capabilities. Where outcomes are documented, the evidence suggests reported progress in student competency development and curriculum integration, though these findings must be interpreted cautiously given the limited scope and inconsistent nature of available assessment materials. The substantial variation in documentation practices suggests that outcome measurement itself remains an evolving institutional capacity, with organizations developing assessment approaches alongside policy implementation rather than following predetermined evaluation frameworks.

**Table 4:** Implementation outcomes based on assessment documentation.

Outcome dimension	Assessment document A (institution A, 2023)	Assessment document B (institution B, 2022)	Quality evaluation report (institution C, 2023)
Student learning outcomes	“Community health competency assessment shows students achieving satisfactory proficiency in preventive care and health promotion skills”	“Student performance in community health modules demonstrates improvement from baseline”	Learning outcome data not included in quality evaluation report
Curriculum effectiveness	“Integration of community health content achieved across core courses with positive faculty feedback”	Curriculum effectiveness measures not documented in available assessment	“Curriculum alignment with national standards verified through external review process”
Faculty development impact	Faculty development outcomes not assessed in available documentation	“Faculty competency in community health education shows improvement as evidenced by teaching evaluations”	Faculty development impact not measured in quality evaluation
Community partnership outcomes	“Student placement experiences in community health settings received favorable evaluation from supervisors”	Community partnership effectiveness not systematically documented	Community partnership outcomes not measured in quality evaluation
Quality assurance compliance	“Provincial quality indicators achieved at satisfactory compliance level with improvements noted in assessment practices”	“Internal quality monitoring demonstrates progress toward policy objectives”	“External quality review confirms institutional compliance with national education standards”
Program sustainability	Sustainability measures not addressed in assessment documentation	Sustainability indicators not documented in implementation assessment	“Program sustainability supported by stable enrollment patterns and partnership engagement”

This table presents implementation outcomes based on available assessment documentation from three institutions, with each column representing a different assessment document. Scope and focus vary considerably across institutions; missing information reflects limited availability of systematic outcome measurement practices. Findings represent preliminary indicators rather than comprehensive effectiveness evaluation.

Further examination indicates that assessment documentation varies not only across institutions but also across outcome dimensions, with institutions at different stages of developing systematic evaluation approaches. Table 4 illustrates this variation, with some outcome dimensions receiving detailed assessment documentation while others remain undocumented across the sampled institutions. The substantial proportion of undocumented outcome areas, combined with the self-evaluative nature of available assessment documentation, underscores the preliminary character of effectiveness indicators presented in this section.

## 5 Discussion and limitations

### 5.1 Discussion

This research contributes to policy transmission theory by identifying an adaptive transmission pattern that challenges linear implementation assumptions. The analysis suggests that the observed policy transmission pattern involves systematic content evolution rather than mechanical replication across governance hierarchies. Contemporary mixed-methods research emphasizes understanding these transmission processes to enhance policy effectiveness (22), while program evaluation literature suggests that implementation success requires recognition of institutional variation and adaptive capacity (23). The observed pattern indicates consistent linguistic evolution from strategic to operational directives, with provincial levels serving as critical intermediary translators converting broad national objectives into contextually relevant institutional guidance.

Institutional response patterns align with broader trends in competency-based medical education reform, where organizations demonstrate varying curriculum modification approaches based on existing capabilities and contextual constraints (19). In a systematized review of 38 CBME evaluation studies, Alharbi (19) found that 84% did not report the evaluation approach or model used, and proposed a micro-meso-macro framework for understanding how institutional capabilities shape curriculum adaptation. The present study provides empirical grounding for this observation by tracing how policy content evolves across governance levels in a specific national context, whereas Alharbi documented the conceptual need for multi-level evaluation without examining the transmission process itself. Moreover, Alharbi’s observation that institutional resource conditions shape the scope and depth of curriculum adaptation resonates with the differential implementation capacities observed across the three institutions examined here. Health humanities and curriculum evaluation research highlights the importance of institutional flexibility in educational transformation processes (24), while systems thinking approaches emphasize adaptive implementation strategies that accommodate organizational complexity (25).

International comparisons suggest similarities between China’s health education policy implementation and reform efforts in other developing countries, particularly regarding challenges of translating national health objectives into institutional practices. A recent analysis of 15 national-level Chinese college mental health education policies using the PMC-Index model identified systematic weaknesses in policy guarantee mechanisms, cross-sectoral coordination, and institutional type adaptability (26). Their finding that national policies struggle to meet the diverse needs of different types of higher education institutions is consistent with the present results, which demonstrate how vocational medical colleges must generate substantial new content to bridge the gap between generic national directives and institution-specific curricular requirements. The present study complements Yu et al.’s policy text analysis with institutional-level examination of how adaptation actually unfolds, capturing transmission mechanisms across governance hierarchies. Mixed-methods approaches provide methodological support for comprehensive policy evaluation strategies (27). The effectiveness of undergraduate clinical education programs depends significantly on institutional capacity to adapt external mandates to local contexts (8), suggesting that the identified transmission mechanisms may have broader applicability beyond the Chinese context. Physical education and health curriculum reform experiences further illustrate the challenges and opportunities inherent in large-scale educational transformation across governance levels (28).

Beyond cross-national patterns, the findings are also consistent with broader challenges identified in interprofessional education and collaborative curriculum development literature, where community health workforce preparation is shaped by institutional capacity and cross-disciplinary coordination (29). Mixed-methods contributions to health policy research demonstrate the importance of examining both process and outcome dimensions in policy evaluation (30), while health policy systems research in developing countries emphasizes comprehensive analytical frameworks that capture implementation complexity (31). The observed institutional responses suggest that community health curriculum reform requires sustained attention to faculty development and community partnership building alongside structural modifications.

These patterns carry practical implications for health workforce development within the “Healthy China 2030” framework, extending beyond medical education to systematic community health capacity building approaches (32). Educational reform implementation research demonstrates the importance of aligning curriculum transformation with broader health system objectives (33), while strategic public health workforce development initiatives emphasize coordinated policy implementation across organizational levels (34). Community health worker development literature supports comprehensive training approaches integrating policy objectives with practical implementation considerations (35).

### 5.2 Limitations

Several limitations warrant consideration when interpreting these findings. The institutional sample (*n* = 3) represents a small fraction of China’s higher vocational medical colleges, and the purposive selection of institutions with relatively complete documentary records may introduce positive reporting bias. Given that all three institutions are located in eastern China, the observed transmission patterns may not generalize to central and western provinces, where resource conditions and institutional contexts differ considerably.

The reliance on document analysis without stakeholder input excludes perspectives central to the “Actors” dimension of Walt and Gilson’s framework. Consequently, the study cannot assess how decision-makers at each governance level shaped transmission outcomes or how frontline educators experienced the reform process. Complementary methodological approaches incorporating stakeholder perspectives would be needed to address these questions.

As noted in Section 3.1, the cross-sectional design precludes assessment of longitudinal policy effects, and the fragmented assessment data documented in Section 4.4 further reflects uneven institutional monitoring capacity. Accordingly, findings should be interpreted as illustrative cases rather than representative patterns. Future research should prioritize multi-province comparative designs, longitudinal tracking of implementation outcomes, and mixed-methods approaches incorporating stakeholder interviews to address the dimensions that document analysis cannot capture.

## 6 Conclusion

This research provides preliminary empirical insights into an adaptive policy transmission pattern that operates through systematic content evolution rather than mechanical policy replication across governance hierarchies. The analysis suggests that “Healthy China 2030” influences community health curriculum reform through three critical transmission mechanisms: content adaptation, language simplification, and contextual integration. These mechanisms are associated with the evolution of national strategic objectives into operational institutional practices while maintaining policy coherence, though the extent of this transformation varies considerably across policy domains and institutional contexts. The study traces how policy language evolves from strategic concepts into concrete operational implementation steps and identifies that efficient transfer appears associated with purposeful facilitation of content creation rather than reliance on standard compliance.

This research advances Walt and Gilson’s policy triangle framework through empirical illustration of its operationalisation in educational policy environments through systematic documentation of cross-level adaptation processes. The findings contribute to understanding of multi-level governance processes by illustrating how institutional difference constitutes adaptive strength rather than implementation weakness, enabling organisations to pursue policy ends through diverse channels of mobilised capability while also conforming to external demands. However, results of implementation remain difficult to assess as a whole due to incomplete institutional assessment routines and the tentative nature of current effectiveness indicators.

Policy makers should design implementation mechanisms anticipating and enabling systematic content creation at various governance levels (as illustrated by the three-level content evolution pattern identified in Section 4.1) while building mechanisms for enhancing institutional assessment capacity (as indicated by the documentation gaps identified in Section 4.4). Successful health workforce development policies require adaptive frameworks accommodating institutional variation and yet enabling strategic coherence, supported by improved systems of outcome measurement (as suggested by the differential institutional responses documented in Section 4.3). Future research should investigate relationships between transmission mechanisms and actual changes in community health service quality, and employ longitudinal mixed-methods designs to capture both the actor dimensions and the temporal dynamics that the present cross-sectional document analysis could not address, providing needed insights to strengthen evidence-based health education policy implementation in developing countries undertaking systematic workforce development initiatives.

## Data Availability

The original contributions presented in the study are included in the article/supplementary material, further inquiries can be directed to the corresponding author/s.
